# Glucose and Fat Tolerance Tests Induce Differential Responses in Plasma Choline Metabolites in Healthy Subjects

**DOI:** 10.3390/nu10091209

**Published:** 2018-09-01

**Authors:** Rima Obeid, Hussain M. Awwad, Astrid Ines Knell, Ulrich Hübner, Jürgen Geisel

**Affiliations:** Department of Clinical Chemistry and Laboratory Medicine, Saarland University Hospital, Building 57, 66424 Homburg/Saar, Germany; ph.hussainawwad@yahoo.com (H.M.A.); s9asknel@stud.uni-saarland.de (A.I.K.); ulrich.huebner@uks.eu (U.H.); juergen.geisel@uks.eu (J.G.)

**Keywords:** choline, trimethylamine N-oxide, diabetes, methylation, metabolic syndrome, lipids, glucose

## Abstract

Plasma choline shows associations with plasma glucose and lipids. We studied changes of choline metabolites after oral glucose tolerance test (OGTT) and fat tolerance test (OFTT). Eighteen healthy subjects (mean age 54.3 years; BMI 26.8 kg/m^2^) underwent 2 tests. First, OFTT (80 g fat) was applied and blood was collected at baseline and 4 h after OFTT. Seven days later, 75 g glucose was applied and blood was collected at baseline and 2 h after OGTT. Plasma concentrations of choline, betaine, trimethylamine N-oxide (TMAO), dimethylglycine, S-adenosylmethionine (SAM), lipids and glucose were measured. After OFTT, plasma choline declined (10.6 to 9.2 µmol/L; *p* = 0.004), betaine declined (33.4 to 31.7 µmol/L; *p* = 0.003), TMAO slightly increased (4.1 to 5.6 µmol/L; *p* = 0.105), glucose declined (5.39 to 4.98 mmol/L; *p* < 0.001), and triglycerides increased (1.27 to 2.53 mmol/L; *p* < 0.001). After OGTT, plasma choline increased (10.1 to 11.1 µmol/L; *p* < 0.001), TMAO declined (4.0 to 3.5 µmol/L; *p* = 0.029), dimethylglycine declined (2.0 to 1.7 µmol/L; *p* = 0.005), SAM declined (103 to 96 nmol/L; *p* = 0.041), but betaine, glucose, and SAM were unchanged. In conclusion, OFTT lowered plasma betaine and choline and caused heterogeneous changes in plasma TMAO. OGTT reduced the flow of methyl groups and plasma TMAO.

## 1. Introduction

Choline is a precursor of phosphatidylcholine and a methyl donor (via irreversible mitochondrial oxidation to betaine). Betaine is provided by the diet or from choline oxidation. Betaine is an important osmolyte and a methyl donor for homocysteine. The methylation reaction mediated by betaine-homocysteine methyltransferase (BHMT) gives dimethylglycine and methionine that is in turn the precursor of the universal methyl donor, S-adenosylmethionine (SAM).

A possible role of choline in cardiometabolic diseases has gained attention in recent years. Postprandial hypertriglyceridemia is associated with cardiovascular risk factors. Choline is required for synthesis of phosphatidylcholine that constitutes an integral part of lipoprotein particles and is thus involved in clearance of fats from blood stream and tissues. Accordingly, high choline intake can abolish the effect of high fat diet on liver steatosis. Plasma concentrations of free choline, betaine, and dimethylglycine show diverse associations with cardiometabolic risk factors [1,2,3]. Low fasting plasma betaine and dimethylglycine (i.e.; a betaine demethylation product) predict incident diabetes [4]. Trimethylamine N-oxide (TMAO), a gut bacteria choline-derived metabolite, is positively associated with cardiovascular risk factors [2], vascular diseases [5], and is elevated in patients with type 2 diabetes [2,6]. In individuals at risk of diabetes, fasting plasma TMAO concentrations show positive correlation with fasting plasma glucose and total- and low-density lipoprotein (LDL)-cholesterol, but not with 2 h-plasma glucose or fasting triglycerides after oral glucose tolerance test (OGTT) [7].

When individuals at risk for diabetes had modified their diet and lifestyle, the changes of plasma TMAO concentration were heterogeneous. A raise of plasma TMAO was associated with improved insulin sensitivity and lowering of plasma lipids [7], suggesting that TMAO elevation in plasma could parallel glucose and/or lipid metabolisms. TMAO is produced from trimethylamine by flavin-containing monooxygenase 3 (FMO3) mainly in the liver. The expression of FMO3 in adipose tissues (but not in the liver) is positively associated with obesity and inversely associated with insulin sensitivity index [8]. Thus, adipose tissues could be a source of TMAO that is associated with enhanced insulin sensitivity.

Plasma betaine, choline, and dimethylglycine are rather stable upon retesting [9,10], whereas plasma TMAO is generally highly variable [9,10]. Ingestion of fish (within 15 min) or beef and egg (after 2–4 h) raises plasma TMAO, but the effect appears to level off 6–12 h later [11,12], suggesting that ≥8 h fasting time before testing TMAO could be sufficient. After six hours of oral ingestion of 50 mg d9-TMAO, only 5.5% of the dose retained in plasma, while 19% was detected in muscle and 42% was excreted in urine [13]. This is in line with studies showing that the major part of the between-individual variations in plasma TMAO in healthy subjects was not explained by possible sources of TMAO in the diet [14]. In general, plasma choline metabolites can be argued to be influenced by liver, kidney, muscle, and adipose tissues metabolism.

Glucose transport across the brush border occurs by a Na^+^/glucose cotransporter. The exit of glucose of the cell across the basolateral membrane occurs through another transporter, GLUT2. In contrast, triglycerides are digested in the stomach, while most fats are hydrolyzed in the duodenum and jejunum and converted into lipid micelles that are partly taken up in the jejunum. A diet that is rich in carbohydrate or fiber can differentially change lipid metabolism by altering intestinal lipase (carbohydrate increases and fiber decreases lipase). Lipid uptake mechanisms (simple passive diffusion or protein-facilitated transport) take place in enterocyte brush border membrane with participation of several receptors and ATP-dependent sterol transporters across the membrane [15]. Postprandial hypertriglyceridemia is ameliorated by several dietary components such as fibers, polyphenols, medium-chain fatty acids, and long-chain n-3 polyunsaturated fatty acids [16]. High fat or glucose intake may impact choline uptake or metabolism.

The objective of the present study was to investigate changes of plasma concentrations of choline metabolites after oral fat tolerance test (OFTT) and OGTT among healthy individuals. We hypothesized that acute consumption of fat or glucose (exposures) affects choline metabolism as indicated by plasma concentrations of choline-derived metabolites (outcome). The oral tolerance tests were applied subsequently within one week apart and within-subject changes of plasma choline metabolites were investigated and compared between the tests.

## 2. Materials and Methods

### 2.1. Study Population

This intervention study investigated the intra-individual changes of plasma concentrations of choline metabolites after ingesting standardized amounts of fat and glucose applied on two independent days with one week in between. The study was conducted at the Department of Clinical Chemistry and Laboratory Medicine, Saarland University Hospital, Germany between May 2016 and January 2017 (ClinicalTrial.gov.ID: NCT02603237).

The inclusion criteria were: adults, men and women, age between 40 and 66 years, and a stable dietary pattern and body weight over the last 6 months. The exclusion criteria were; pregnant women, BMI > 25 kg/m^2^, using multivitamin supplements, diseases known to influence choline metabolism; diabetes (type 1 or 2), hypertension, malabsorption disorders, liver or renal disorders, and drugs (i.e.; anti-depressants, anti-epileptics, anti-folate, fibrate, statins, sex hormones, or metformin). Among the 20 subjects who were initially interested in participation; one subject was older than 66 and had multi-morbidities (cancer, vascular disease) and another one refused to participate in the OFTT test. Eighteen individuals (8 men; 10 women) took part in the study and attended the 2 visits.

The protocol was reviewed and approved by the Ethical Commission of the Saarland Region (approval Nr. 256/15). The study was conducted according to the ethical principles documented in Helsinki Declaration and all subjects provided signed informed consents to the study.

### 2.2. Study Design

The study consisted of two visits with a washout phase of approximately 7 days between the two tests (Supplemental Figure S1). All participants received OFTT in the first visit in order to determine their fasting glucose and glycated haemoglobin (HbA1c) before applying OGTT in the second visit. Subjects who had undiagnosed type 2 diabetes had to be excluded before applying OGTT in the second visit. Thus, the study was not planned to follow a classical randomized design.

During visit 1, blood and urine samples were collected in the morning after an overnight fasting (≥8 h). Then, participants ingested 250 g milk cream that provided 80 g fat (32% fat, Landliebe^®^) within 5 min. The nutritional composition of the milk cream is shown in Supplemental Table S1. Blood and urine samples were collected again 4 h after OFTT. The participants were asked not to consume any solid foods during the 4 h, while drinking water was allowed during this period. During the second visit, subjects with both normal fasting plasma glucose and HbA1c, as a result of first visit, underwent OGTT. Fasting blood samples were collected before applying OGTT and a second blood collection was conducted 2 h after drinking 300 mL syrup containing 75 g glucose (ACCU CHEK, Dextro O.G-T.; Roche^®^, Basel, Switzerland).

Information was collected on health history, weight, height, and waist-to-hip ratio (measured). The participants (*n* = 18) were all found to be free of diabetes during visit 1 and were thus able to take part in the OGTT test.

### 2.3. Blood and Urine Collection

Venous blood was collected into heparin- and EDTA-containing tubes before applying OFTT and OGTT. Those samples are referred to as baseline samples, which were collected after an overnight fasting (≥8 h). Additional blood samples were collected 4 h after OFTT and 2 h after OGTT. Blood count was directly conducted in EDTA whole blood. Blood samples were centrifuged and immediately separated into aliquots and stored at −70 °C. After centrifugation, EDTA-plasma (500 µL) was immediately acidified by adding 50 µL acetic acid (1N) and acidified samples were stored at −70 °C for measurements of SAM and S-adenosylhomocysteine (SAH).

Urinary samples (morning urine at baseline and after 4 or 2 h of applying the tests) were collected and separated into aliquots that were stored at −70 °C until analysis. Concentrations of all markers were measured within 8 months after sample collection. Concentrations of choline biomarkers were measured in all samples (4 plasma or urine samples for each participant) from the same person in the same run to reduce analytical variations.

### 2.4. Measurements of Choline and Its Metabolites

Urine samples were thawed and centrifuged at 10,000× *g* for 5 min before measurement of creatinine or choline biomarkers. Plasma and urinary concentrations of TMAO and trimethylamine (a direct gut bacteria metabolite and a TMAO precursor) were measured using ultraperformance liquid chromatography-tandem mass spectrometry (UPLC-MS/MS) with isotope labeled internal standards (d_9_-TMAO and d_9_-trimethylamine) as described before [17]. Briefly, methanol/acetonitrile mix (15:85) containing 0.2% formic acid was added to the sample. The internal standards were added and samples were mixed, centrifuged for 5 min at 10,000× *g*, and then separated on an Acquity UPLC BEH HILIC column (100 × 2.1 mm) with an Acquity HILIC VanGuard pre-column (5 × 2.1 mm) (Waters Corporation, Milford, MA, USA). The column temperature was set to 30 °C and the flow rate was 0.4 mL/min. The gradient elution consisted of 15 mmol/L ammonium formate (solvent A, pH = 3.5) and acetonitrile (solvent B). Positive multiple reaction monitoring mode was used and TMAO and TMA were monitored using the following ion transitions: *m*/*z* 75.90→58.10 and 59.98→44.02, respectively. d_9_-TMAO and d_9_-TMA were monitored at *m*/*z* 84.90→66.05 and 129.77→48.09, respectively. Urine TMA and TMAO were prepared using the same method after diluting the sample 1:5 in distilled water. The between-day coefficients of variation (CV) of TMAO and trimethylamine were <8% and <14%, respectively. Plasma trimethylamine levels were below the assay low detection limit (LOD = 0.12 µmol/L) in all 71 samples out of the 72 sample tested (1 sample had 0.19 µmol/L). Thus, the results of plasma trimethylamine are not presented as numerical values.

Plasma and urine concentrations of choline, betaine, and dimethylglycine in addition to plasma SAM, and SAH were measured using UPLC-MS/MS technique and established methods [18,19]. Choline, betaine, and dimethylglycine were separated on an Acquity UPLC BEH HILIC column (100 mm × 2.1 mm (i.d.); 1.7 µm particle size) with an Acquity HILIC VanGuard pre-column (5 mm × 2.1 mm (i.d.); 1.7 µm particle size) and a 0.2 µm in-line filter (Waters Corporation). The column temperature was 30 °C and the flow rate was 0.6 mL/min. The solvent A and B were 15 mmol/L ammonium formate (pH 3.5) and acetonitrile, respectively. d_9_-Betaine chloride, d_9_-choline chloride (Isotec, Sigma-Aldrich, Munich, Germany), and d_6_-dimethylglycine HCl (CDN isotopes, Simport, QC, Canada) were used as internal standards. Plasma, or urine (diluted in H_2_O 1:5) samples were added to the internal standard mix in acetonitrile. After protein precipitation and vortexing, the samples were centrifuged and 1 µL was injected into the UPLC-MS/MS.

Concentrations of SAH and SAM were measured using labelled isotopes as internal standards (^13^C_5_-SAH and d_3_-SAM). Sample cleanup was conducted using solid-phase extraction (SPE) columns containing phenylboronic acid (Varian Bond Elut PBA columns, Varian Inc., Palo Alto, CA, USA). Aqueous acetic acid, pH 2.636 was used to elute the analytes. The chromatography separation was conducted using ACQUITY UPLC BEH C18 Column, 130 Å, 1.7 µm, 2.1 mm × 50 mm and ACQUITY UPLC BEH C18 VanGuard Pre-column, 130 Å, 1.7 µm, 2.1 mm × 5 mm. The column temperature was maintained at 30 °C, with a mobile phase flow rate of 0.35 mL/min. The mobile phase consisted of 100% aqueous acetic acid (glacial), pH 2.636. The injected volume was 10 µL.

EDTA whole blood HbA1c concentrations were measured using an automated VARIANT™ II TURBO system (Bio-Rad Laboratories, Munich, Germany). Plasma and urinary creatinine and plasma glucose, lipids (high density lipoprotein, low density lipoprotein, triglyceride and total cholesterol), and liver markers such as aspartate-amino-transferase (AST) and alanine-amino-transferase (ALT) were measured using automated methods (COBAS INTEGRA System, Roche Diagnostics, Germany) at the central laboratory of the Saarland University Hospital. Plasma lipids were measured only before and after OFTT (in the first visit).

### 2.5. Statistical Analyses

Sample size calculation was based on potential changes in plasma concentrations of TMAO (pre-post levels compared with paired *t*-test). We considered a change (increase or decrease) of approximately 50% from baseline (approximately 2 µmol/L change compared with zero change) as metabolically relevant. Assuming a standard deviation of the change of (approximately 2 µmol/L), a power of 0.95, and α = 0.05, we estimated a sample size of 13 participants undergoing each test would be sufficient. Changes in betaine, choline, dimethylglycine, and SAM were not used for sample size estimation because the extent of the change could not be postulated based on previous studies. Stratification according to sex was not initially planned because we found no differences in TMAO between men and women in an earlier study [2]. However, posthoc explorative analyses of the results according to sex are shown in Supplemental Tables S4 and S5.

Continuous variables were tested for normal distribution using Kolmogorov-Smirnov test and Lilliefors Significance Correction. The following variables showed skewed distribution: TMAO before OFTT and dimethylglycine before OGTT. TMAO values (both before and after OFTT) were log-transformed and were both found to be normally distributed after the transformation. Log-transformed TMAO was used for paired comparisons (before and after OFTT). The log-transformed dimethylglycine before OGTT was not normally distributed and the non-transformed values were compared. Results of continuous variables and changes over time are shown as mean ± SD (standard deviation). The within-individual differences in concentrations of the biomarkers (pre versus post) were analyzed by using paired-t-test. Changes of the choline metabolites were calculated as; deltaOFTT after OFTT = post-OFTT levels − pre-OFTT levels and deltaOGTT after OGTT = post-OGTT levels − pre-OGTT levels. DeltaOFTT% is the post-OFTT levels − pre-OFTT levels*100/pre-OFTT levels and deltaOGTT % is the post-OGTT levels − pre-OGTT levels × 100/pre-OGTT levels. Paired-*t*-test was used to compare changes after OFTT (deltaOFTT) with those after OGTT (deltaOGTT). Fasting concentrations of the metabolites before OFTT were compared with those before OGTT (measured one week later) by using paired-*t*-test.

One-way analysis of variance (ANOVA) test was used to compare all continuous variables between men and women. Stepwise multiple linear regression analyses were applied to study the predictors of plasma TMAO, glucose, and triglycerides after OFTT and plasma TMAO and glucose after OGTT.

Statistical analyses were conducted using IBM SPSS 24.0 (IBM Corp.; Armonk, NY, USA). *p* values below 0.05 were considered statistically significant and those between 0.05 and 0.10 were interpreted as showing a tendency.

## 3. Results

The study included 18 participants with a mean age of 54.3 (SD = 5.7) years. Men and women showed significant differences in waist-to-hip ratio (0.99 vs. 0.87), age (57.5 vs. 51.5 years), plasma creatinine (89.5 vs. 72.9 µmol/L), and blood haemoglobin (15.4 vs. 13.4 g/dL) (Table 1).

### 3.1. Levels of Plasma and Urine Biomarkers Following OFTT

Table 2 shows concentrations of choline related metabolites and lipids before applying OFTT (baseline or 8 h fasting levels) and 4 h after OFTT. Plasma concentrations of choline were significantly lower after OFTT compared with the baseline concentrations (9.2 vs. 10.6 µmol/L; *p* = 0.004). Moreover, the concentrations of betaine declined (33.4 to 31.7 µmol/L; *p* = 0.003), while the concentrations of TMAO tended to increase (4.1 to 5.6 µmol/L: *p* = 0.105) after OFTT. Choline/betaine ratio declined (0.33 to 0.30; *p* = 0.010) and choline/TMAO ratio declined (3.08 to 2.48; *p* = 0.025). Plasma concentrations of dimethylglycine, SAM, SAH, and total cholesterol did not differ significantly between baseline and after OFTT. Plasma concentrations of glucose declined (5.39 to 4.98 mmol/L; *p* < 0.001), and those of triglycerides increased (1.27 to 2.53 mmol/L; *p* < 0.001).

The concentrations of TMAO tended to increase after OFTT in the whole group. TMAO declined (range −3% to −25% from baseline) in 9 participants (6 women, 3 men) after OFTT, while it increased (range +3% to +408%) in 9 subjects (4 women, 5 men). We compared subjects who showed an increase in plasma TMAO with those who showed a decrease in TMAO after OFTT. No differences were detected in age, BMI, W/H ratio, or concentrations of any of the measured biomarkers in plasma.

Baseline urinary excretions of betaine (8.0 vs. 8.1 µmol/mmol creatinine), choline (2.6 vs. 2.6 µmol/mmol creatinine), dimethylglycine (2.0 vs. 1.8 µmol/mmol creatinine) and TMAO (47.2 vs. 48.5 µmol/mmol creatinine) were not significantly different from urinary excretions after OFTT (Supplemental Figure S2). Urinary trimethylamine excretion increased after OFTT compared with baseline levels (mean 0.128 vs. 0.234 µmol/mmol creatinine; *p* = 0.027).

### 3.2. Levels of Plasma and Urine Biomarkers Following OGTT

Plasma concentrations of the metabolites before and 2-h after OGTT are shown in Table 3. In contrast to the changes seen after OFTT, plasma choline increased after OGTT (10.1 to 11.1 µmol/L: *p* < 0.001), TMAO declined (4.0 to 3.5 µmol/L: *p* = 0.029), choline/betaine ratio increased (0.30 to 0.34; *p* = 0.001), and choline/TMAO ratio increased (3.3 to 4.1: *p* < 0.001). Plasma concentrations of dimethylglycine declined (2.0 to 1.7 µmol/L: *p* = 0.005), SAM declined (103 to 96 nmol/L; *p* = 0.041), while betaine, glucose, and SAH remained unchanged after OGTT (Table 3). After OGTT, plasma TMAO declined in 15 individuals (range −5% to −39%), while it increased in 3 individuals (range 4 to 39%).

Multiple regression analyses were applied to predict post-OFTT TMAO, glucose, and triglycerides and post-OGTT TMAO and glucose (Supplemental Table S2). In general, baseline plasma concentrations of TMAO were the main predictor of the post-tests TMAO levels. However, when delta-TMAO was entered as a dependent variable, none of the measured variables was a significant predictor of delta-TMAO.

Urinary excretions of betaine (7.1 vs. 8.3 µmol/mmol creatinine; *p* = 0.007) and choline (2.6 vs. 3.4 µmol/mmol creatinine) were higher after OGTT compared with baseline concentrations. In contrast, urinary dimethylglycine (1.5 vs. 1.8 µmol/mmol creatinine), trimethylamine (0.11 vs. 0.10 µmol/mmol creatinine) and TMAO (40.7 vs. 38.4 µmol/mmol creatinine) after OGTT did not differ significantly from baseline levels (Supplemental Figure S2).

### 3.3. Comparative Analysis of Choline Metabolites Following OFTT and OGTT Tests

In order to compare within-subject changes of choline metabolites caused by OFTT and those caused by OGTT in the same individuals, we first compared baseline plasma concentrations measured within 1 week. No significant differences were found between baseline plasma concentrations of the choline markers measured before OFTT and before OGTT (Supplemental Table S3).

Changes of plasma levels from baseline after OFTT (deltaOFTT) were compared with changes of the levels after OGTT (deltaOGTT). We observed significant differences in changes of choline (declined after OFTT and increased after OGTT; *p* < 0.001). Changes of the following markers showed a tendency to differ between the two tests: TMAO increased after OFTT, but declined after OGTT (*p* = 0.050); betaine declined after OFTT, but did not change after OGTT (*p* = 0.057); and dimethylglycine declined to a stronger degree after OGTT than after OFTT (*p* = 0.093) (Supplemental Figure S3). When the percentage changes were compared after OFTT with those after OGTT, delta choline% and delta betaine % were significantly different, and delta TMAO % tended to be different between OFTT and OGTT (*p* = 0.057) (Supplemental Figure S3).

### 3.4. Comparative Analysis of Choline Metabolites according to Sex

Men had higher plasma choline, betaine, and SAH concentrations compared to women (Supplemental Tables S4 and S5). These differences were consistently detected before and after OFTT and OGTT. After OFTT, plasma choline tended to decline or declined (*p* = 0.132 in men and *p* = 0.010 in women) and plasma betaine declined in both sexes (*p* = 0.057 in men, and *p* = 0.025 in women). No other significant sex-specific changes were detected after OFTT. After OGTT, plasma choline increased (*p* = 0.011 in men, and *p* = 0.007 in women), plasma dimethylglycine declined or tended to decline (*p* = 0.027 in men, *p* = 0.114 in women), and plasma TMAO declined or tended to decline (*p* = 0.006 in men, *p* = 0.113 in women). The individual changes of plasma TMAO levels according to the test and sex are shown in Supplemental Figure S4. In general, stratification according to sex tended to weaken the significance of the results possibly due to a smaller sample size. Although men and women differed in their plasma concentrations of some metabolites such as choline and betaine, the changes of any metabolite observed after OFTT or OGTT (calculated as post-pre values) were not proportional to the baseline levels of the same metabolite.

## 4. Discussion

We reported significant within-individual changes in plasma concentrations of choline metabolites after OFTT and OGTT in healthy individuals (Supplemental Figure S5). Changes of plasma choline and TMAO from baseline were in opposite directions; plasma choline declined and TMAO increased after OFTT, while choline increased and TMAO declined after OGTT. Men and women had different betaine and choline concentrations, but the changes of choline metabolites after the tests were not related to baseline concentrations and not explained by sex. These results are likely to represent a metabolic adaptation to acute ingestion of glucose or fat (postprandial hypertriglyceridemia).

Chronic consumption of fats and/or glucose is associated with multi-components of the metabolic syndrome such as, diabetes, abdominal obesity, and hypercholesterolemia [20]. Healthy none-obese subjects responded to OGTT by sparing choline and betaine as methyl donors (increased urinary excretion of betaine and choline and increased plasma choline), maintaining glucose, while plasma concentrations of dimethylglycine and SAM declined. Therefore, OGTT reduced the flow of methyl groups via BHMT that transfers a methyl group to produce dimethylglycine and methionine. Urinary excretion of betaine and choline were increased after OGTT, thus supporting the downregulation of the methyl flow through the BHMT pathway. Downregulation of methyl groups has been reported after experimental exposure to high concentrations of glucose [21]. Chen et al. have shown that intracellular concentrations of choline are elevated and those of dimethylglycine are lowered in neural PC12 cells exposed to high glucose media [21]. Dimethylglycine is metabolized to glycine. Plasma levels of dimethylglycine and glycine show an inverse correlation with fasting glycemia and insulin resistance in human studies [22,23]. Ho et al. performed metabolic profiling in 377 subjects without diabetes before and after OGTT [24]. Plasma levels of methionine (−1.44 fold), and glycine (−1.15 fold) declined after OGTT [24]. As in our study, dimethylglycine declined after OGTT (−1.06 fold) [24]. Combined with our results, lowering methionine, dimethylglycine, glycine, adenosine mono- and di-phosphate and several purines after OGTT in the study of Ho et al.; strongly suggests that the methylation pathway is down regulated in healthy people under glucose challenge [24]. It is possible that glycine (and formate) pathway links methyl groups and glucose oxidation by regulating mitochondrial flavin-adenine dinucleotide (FAD) cofactors. FMO3 (UniProt P31513) interacts with glucose metabolism [25]. Flavin mononucleotide is down regulated after OGTT [24] which may lead to a transient reduction in FMO3 activity and may explain our results on lowering TMAO after OGTT in healthy subjects. In contrast, TMAO is elevated in manifested diabetes which could reflect failure in controlling blood glucose, adaptation to chronically elevated blood glucose, or enhanced FMO3 expression in adipose tissue of patients with diabetes [8].

Concentrations of dimethylglycine and SAM remained unchanged 4 h after OFTT, suggesting that betaine (declined after OFTT) had been utilized to maintain sufficient methyl groups as suggested by unchanged dimethylglycine and SAM in plasma. After OFTT, plasma choline declined, while triglycerides increased. Postprandial hypertriglyceridemia may enhance choline conversion into phosphatidylcholine that is required to solubilize chylomicrons and very low density lipoprotein-(VLDL) particles. An SAM-dependent phosphatidylethanolamine methyl transferase (PEMT) is an important source of phosphatidylcholine in the liver. PEMT regulates lipid homeostasis under high fat diet [26,27] and it could be theoretically activated under a high fat diet, thus leading to higher utilization of betaine and choline as methyl donors.

Eating egg yolk, fish, or beef is associated with an increase in plasma TMAO in healthy subjects [11,28]. Fruits caused a reduction in plasma TMAO by 0.4 µmol/L. According to our results, this could be related to a high glycemic index [11]. Divergent changes of plasma TMAO have been previously reported among healthy volunteers (TMAO-producers and non-producers) [11]. Although, the overall effect in our study was a trend towards an increase in plasma TMAO after OFTT, however, TMAO declined after OFTT in 9 out of 18 participants, while it increased in 9 participants. These opposite changes remained unexplained, but might be due to differences in gut bacteria [11]. In contrast, the increase of TMAO after OGTT occurred in 15 out of 18 subjects. Suppression of FMO3 expression in animals caused weight reduction, especially due to lowering white adipose tissue weight and increasing lean mass [8]. Moreover, administration of TMAO to mice fed a high fat diet improved glucose tolerance [29]. In our study, the diverse response of TMAO after OFTT could be related to lean body mass that is not reflected by BMI or waist-to-hip ratio.

Long term effects of dietary patterns and controlled dietary intakes of fat and sugars on choline metabolism are not well studied. A positive association between dietary fat intake and plasma betaine has been reported in a study of middle aged and elderly men and women [30]. In young none-obese men consuming a high-fat diet (55% of the calories as fat) for 5 days, intervention with a high fat meal (63% of the calories as fat) caused an increase in plasma TMAO [31]. Boutagy et al. reported an increase in plasma TMAO [31] similar to that found in the present study after OFTT (93% of the calories as fat). Additionally, an increase in plasma TMAO after 9 months of lifestyle modifications has been related to improved insulin sensitivity and lipid profile in an interventional study [7]. A raise in plasma TMAO after OFTT in 9 participants could reflect a high fat dietary pattern while, a decline in TMAO after OFTT in the other 9 participants could reflect a dietary pattern that is rather low in fat. The influence of the diet was not verified in the present study.

Alternative explanations of the current results deserve consideration. It is possible that OGTT and OFTT cause differential alterations in nutrient transport through the gut. Changes in plasma metabolites could be due to excretion of choline and TMAO in the bile and the re-absorption of these compounds or could depend on the osmotic pressure in the gut or differ between fat and sugar milieus. Since urine samples were collected 2 h after OGTT and 4 h after OFTT, we could have missed the time window for collecting urine samples that show significant changes. Similarly, we could have missed an optimal time windows (maximal exposure effect) to collect blood samples. This issue could have influenced the comparisons between concentrations of the biomarkers 2 h after OGTT versus those of 4 h after OFTT. However, this explanation is unlikely because our results after OGTT and OFTT are in agreement with previous studies [24,31]. Limitations of the present study include lack of information on dietary intake or stool microbiome pattern and the lack of standardization of the meals before the tests and the water drinking volume during the test. Also, the limited number of participants could have hided some effects. A post-hoc sample size calculation based on the observed values showed that 33 and 13 subjects would be sufficient to show changes in TMAO after OFTT and OGTT, respectively (Supplemental Table S6). 

In summary, the study has shown that OGTT caused a reduction in plasma TMAO and the methylation markers, dimethylglycine and SAM, increased plasma choline, and caused no change in plasma betaine. In contrast, OFTT caused heterogeneous changes in plasma TMAO, caused no change in plasma methylation markers, lowered plasma levels of choline, betaine, and glucose, and increased postprandial triglycerides.

## 5. Conclusions

Acute ingestion of glucose in healthy subjects lowered the flow of methyl groups and increased plasma choline. Whereas, acute ingestion of fat lowered plasma concentrations of choline and betaine suggesting that both compounds were utilized for producing phosphatidylcholine that is involved in clearance of postprandial triglycerides. The heterogeneous changes in TMAO after OFTT (increased in 50% and declined in 50% of the participants) could reflect differences in metabolic, gut bacterial patterns, or adipose tissue and lean mass, but this needs further investigations.

## Figures and Tables

**Table 1:** Main characteristics of the studied participants.

	All	Men	Women	*p* ^1^
*n*	18	8	10	
Age, years	54.3 ± 5.7	57.9 ± 4.9	51.5 ± 4.7	0.014
BMI, kg/m^2^	26.8 ± 5.2	26.0 ± 3.9	27.4 ± 6.2	0.611
Waist/hip ratio	0.92 ± 0.10	0.99 ± 0.07	0.87 ± 0.09	0.008
Creatinine, µmol/L	80.3 ± 15.0	89.5 ± 13.3	72.9 ± 12.3	0.014
GFR, mL min^−1^ 1.73 m^2^	83 ± 15	82 ± 14	84 ± 16	0.813
CRP, mg/L	2.2 ± 2.9	2.6 ± 4.0	1.9 ± 3.2	0.612
HbA1c, mmol/mol	36 ± 3	35 ± 3	37 ± 3	0.300
AST, UL	25.1 ± 5.9	24.6 ± 6.1	25.6 ± 6.1	0.741
ALT, UL	29.4 ± 11.5	27.4 ± 8.4	31.1 ± 13.7	0.510
Gamma GT, UL	37.1 ± 25.9	45.1 ± 30.4	30.7 ± 21.0	0.251
Total cholesterol, mmol/L	5.52 ± 0.95	5.42 ± 0.63	5.60 ± 1.17	0.700
HDL-cholesterol, mmol/L	1.66 ± 0.36	1.60 ± 0.29	1.70 ± 0.43	0.593
LDL-cholesterol, mmol/L	3.54 ± 0.91	3.48 ± 0.61	3.59 ± 1.14	0.801
Haemoglobin, g/dL	14.3 ± 1.2	15.4 ± 0.81	13.4 ± 0.6	<0.001

Data are shown as mean ± SD. ^1^ *p* values are according to independent *t*-test. Conversion factor for SI units for creatinine: 88.4 µmol/L = 1 mg/dL; total-, HDL- and LDL-cholesterol 1 mmol/L = 38.61 mg/dL. AST, aspartate-amino-transferase; ALT, alanine-amino-transferase; BMI, body mass index; CRP, C-reactive protein; GFR, glomerular filtration rate; gamma GT, gamma-glutamyltranspeptidase; HbA1C, glycated haemoglobin; HDL, high density lipoprotein; LDL, low density lipoprotein.

**Table 2:** Plasma concentrations of choline metabolites, glucose, and lipids at baseline and after applying OFTT during the first study visit.

Plasma Biomarkers	Baseline OFTT (Fasting)	After OFTT (4 h)	Delta_OFTT_^1^ Absolute Changes	Percentage Change from Baseline (%OFTT) ^2^	*p* ^3^
Free choline, µmol/L	10.6 ± 2.3	9.2 ± 2.3	−1.5 ± 1.9	−13 ± 16	0.004
Betaine, µmol/L	33.4 ± 9.2	31.7 ± 9.3	−1.7 ± 2.1	−5.2 ± 6.1	0.003
Dimethylglycine, µmol/L	2.0 ± 0.9	1.9 ± 0.9	−0.05 ± 0.30	−3 ± 25	0.527
Trimethylamine ^4^, µmol/L	<LOD	<LOD	/	/	/
TMAO ^5^, µmol/L	4.1 ± 2.3	5.6 ± 4.1	+1.5 ± 3.7	+41 ± 103	0.105
Choline/betaine ratio	0.33 ± 0.09	0.30 ± 0.08	/	/	0.010
Choline/TMAO ratio	3.08 ± 1.26	2.48 ± 1.69	/	/	0.025
SAM, nmol/L	100 ± 17	99 ± 16	−1 ± 7	−0.6 ± 7.2	0.561
SAH, nmol/L	17.0 ± 4.9	16.1 ± 6.7	−1 ± 5.2	−4.2 ± 37.0	0.465
Glucose, mmol/L	5.39 ± 0.44	4.98 ± 0.35	−0.41 ± 0.32	−7.3 ± 5.8	<0.001
Cholesterol, mmol/L	5.52 ± 0.95	5.57 ± 0.99	+0.05 ± 0.19	+1 ± 3	0.251
Triglycerides, mmol/L	1.27 ± 0.59	2.53 ± 1.32	+1.26 ± 0.84	+101 ± 60	<0.001

Data are mean ± SD. *N* = 18 individuals participated in this test. ^1^ Delta_OFTT_ = (concentrations post OFTT − fasting concentrations before OFTT). ^2^ Percentage change from baseline = (concentrations post OFTT − concentrations at baseline) × 100/baseline concentrations. ^3^ *p* values are according to paired *t*-test. ^4^ The low detection limit (LOD) for trimethylamine in plasma is 0.12 µmol/L. ^5^ TMAO concentrations were log-transformed before applying *t*-test. Conversion factors to SI units: glucose 1 mmol/L = 18.02 mg/dL; cholesterol 1 mmol/L = 38.61 mg/dL; triglycerides 1 mmol/L = 88.5 mg/dL. The baseline levels are the fasting levels. OFTT, oral fat tolerance test; TMAO, trimethylamine N-oxide.

**Table 3:** Plasma concentrations of choline metabolites and glucose at baseline and after applying oral glucose tolerance test in the second study visit.

	Baseline OGTT (Fasting)	After OGTT (2 h)	Delta_OGTT_ Absolute Changes	Percentage Change from Baseline (%_OGTT_)	*p*
Free choline, µmol/L	10.1 ± 2.5	11.1 ± 2.7	1.07 ± 0.92	+11.3 ± 9.9	<0.001
Betaine, µmol/L	34.3 ± 9.8	34.3 ± 9.7	−0.05 ± 2.88	+0.14 ± 7.6	0.943
Dimethylglycine, µmol/L	2.0 ± 0.8	1.7 ± 0.8	−0.32 ± 0.40	−15.8 ± 20.7	0.005
Trimethylamine, µmol/L	<LOD	<LOD	/	/	/
TMAO, µmol/L	4.0 ± 2.1	3.5 ± 2.1	−0.46 ± 0.82	−10.9 ± 17.0	0.029
Choline/betaine	0.30 ± 0.08	0.34 ± 0.09	/	/	0.001
Choline/TMAO ratio	3.3 ± 2.4	4.1 ± 2.7	/	/	<0.001
Glucose, mmol/L	5.36 ± 0.42	4.94 ± 1.13	−0.42 ± 1.23	−7.2 ± 22.0	0.162
SAM, nmol/L	103 ± 18	96 ± 17	−7 ± 13	−5.8 ± 10.9	0.041
SAH, nmol/L	15.4 ± 5.3	14.6 ± 5.3	−0.8 ± 3.5	−3.3 ± 24.4	0.347

Data are mean ± SD. *N* = 18 individuals participated in this test. *p* values are according to paired *t*-test. Delta = (concentrations post OGTT − concentrations at baseline). Percentage change from baseline = (concentrations post OGTT − concentrations at baseline) × 100/concentrations at baseline. The low detection limit (LOD) for trimethylamine in plasma is 0.12 µmol/L. Conversion factors to SI units: glucose 1 mmol/L = 18.02 mg/dL. The baseline levels are the fasting levels. OGTT, oral glucose tolerance test; TMAO, trimethylamine N-oxide.

## Supplementary Materials

The following are available online at http://www.mdpi.com/2072-6643/10/9/1209/s1, Supplemental Figure S1: Study flow diagram, Figure S2: Mean and 95% CI of urinary concentrations of the metabolites (all are µmol/mmol creatinine) before and after OFTT and OGTT. Urinary trimethylamine increased from baseline after OFTT, and urinary betaine and choline increased after OGTT compared with fasting concentrations. Urine samples were collected before OFTT and OGTT, 4 h after OFTT, and 2 h after OGTT. Concentrations of betaine and choline in urine were higher after OGTT as compared with those after OFTT, Figure S3: (A) DeltaOFTT and deltaOGTT of plasma concentrations of betaine, choline, dimethylglycine, and TMAO. The delta was calculated as (post-test concentrations − pre-test concentrations). (B) The percentage changes of the metabolic markers from baseline. The percentage changes were calculated as: (post-OFTT levels − pre-OFTT levels)*100/pre-OFTT levels or (post-OGTT levels − pre-OGTT levels)*100/pre-OGTT levels. *p* values are according to paired *t*-test, Figure S4: Individual TMAO levels (in µmol/L) measured at baseline (before) and after OFTT or OGTT separated according to sex, Figure S5: Summary of the significant changes (or tendencies up to ≤0.10) in plasma metabolites after applying oral fat tolerance test and oral glucose tolerance test, Table S1: Nutritional values of the milk cream (Landliebe^®^) presented in 100 g cream (as declared by the manufacturer), Table S2: Stepwise multiple regression analysis was applied to find predictors of plasma glucose, triglycerides and TMAO following OGTT or OFTT, Table S3: Comparisons of baseline (8 h fasting) plasma metabolites measured before applying OFTT and before applying OGTT (one week later), Table S4: Plasma concentrations of choline metabolites, glucose, and lipids before and after applying OFTT according to sex, Table S5: Plasma concentrations of choline metabolites, glucose, and lipids before and after applying OGTT according to sex, Table S6: Post-hoc calculations of the sample size needed to show significant differences in TMAO according to the mean and SD observed in the present study.
