# Supplementary material for: Glucose and Fat Tolerance Tests Induce Differential Responses in Plasma Choline Metabolites in Healthy Subjects
Source: Nutrients. 2018 Sep 1;10(9):1209. doi: 10.3390/nu10091209 (PMC6165136; doi:10.3390/nu10091209)
Supplement: Supplementary file 1 [file nutrients-10-01209-s001.pdf]

Glucose and Fat Tolerance Tests Induce Differential Responses in Plasma Choline Metabolites in Healthy Subjects

Supplemental Table 1. Nutritional values of the milk cream (Landliebe®) presented in 100 g cream (as declared by the manufacturer).

Total energy	1298 kj
Total calories	310 kcal
Protein	2.4 g (3% of total energy)
Carbohydrates	3.2 g (4% of total energy)
of these sugar	3 g
Fats as unsaturated fatty acids	32 gr (93% of total energy)<br>21.0 gr
Fiber	0
Water	55%

Supplemental Table 2. Stepwise multiple regression analysis was applied to find predictors of plasma glucose, triglycerides and TMAO following OGTT or OFTT.

Dependent variable	Adjusted R^2	Constant	Independent variables with a significant influence	Beta coefficient (95%CI)	*p*
Plasma glucose after OGTT, mmol/L	0.607	15.9	HDL-cholesterol, mmol/L	−2.59 (−3.75,−1.42)	<0.001
			W/H ratio	−7.3 (−11.5,−3.1)	0.002
Plasma TMAO after OGTT, µmol/L	0.843	−0.237	TMAO pre-OGTT, µmol/L	0.94 (0.72,1.15)	<0.001
Plasma triglycerides after OFTT, mmol/L	0.861	0.107	Triglycerides pre-OFTT, mmol/L	1.92 (1.45,2,39)	<0.001
			Cholesterol pre-OFTT, mmol/L	0.31 (0.02,0.59)	0.037
			GFR, ml. min^{-1}. $1.73m^2$	−0.020 (−0.038,−0.002)	0.029
Plasma glucose after OFTT, mmol/L	0.582	2.06	Glucose pre-OFTT, mmol/L	0.55 (0.29,0.82)	<0.001
			SAH pre-OFTT, nmol/L	−0.029 (−0.052,−0.005)	0.020
Plasma TMAO after OFTT, µmol/L (log)	0.673	0.321	TMAO pre-OFTT, µmol/L (log)	1.14 (0.70,1.58)	0.004
			Dimethylglycine pre-OFTT, µmol/L	−0.16 (−0.26,−0.06)	<0.001

All linear regression models included the following independent variables: sex (men = 0, women = 1), age, BMI, waist/hip ratio, in addition to concentrations of all plasma baseline markers that were measured before applying the corresponding tolerance test.

Supplemental Table 3. Comparisons of baseline (8 hrs fasting) plasma metabolites measured before applying OFTT and before applying OGTT (one week later).

	Before OFTT (baseline)	Before OGTT (baseline)	*p*
Free choline, µmol/L	10.6 (2.3)	10.1 (2.5)	0.139
Betaine, µmol/L	33.4 (9.2)	34.3 (9.8)	0.585
Dimethylglycine, µmol/L	2.0 (0.9)	2.0 (0.8)	0.955
TMAO, µmol/L	4.1 (2.3)	4.0 (2.1)	0.794
Glucose, mmol/L	5.39 (0.44)	5.36 (0.42)	0.125
SAM, nmol/L	100 (17)	103 (18)	0.385
SAH, nmol/L	17.0 (4.9)	15.4 (5.3)	0.170

Data are mean (SD). N = 18 individuals participated in this test. P values are according to paired t-test. Conversion factors to SI units: glucose 1 mmol/L = 18.018 mg/dl OGTT, oral glucose tolerance test; OFTT, oral fat tolerance test; SAH, S-adenosylhomocysteine; SAM, S-adenosylmethionine; TMAO, trimethylamine N-oxide.

Supplemental Table 4. Plasma concentrations of choline metabolites, glucose, and lipids at baseline and after applying OFTT according to sex.

	In men			In women				
Plasma biomarkers	Baseline OFTT (fasting)	After OFTT (4 hrs)	*p* [1]	Baseline OFTT (fasting)	After OFTT (4 hrs)	P [1]	P [2] between sex (baseline)	*p* [2] between sex (after OFTT)
Free choline, µmol/L	12.0±1.6	10.9±2.2	0.132	9.5±2.1	7.8±1.3	0.010	0.014	0.001
Betaine, µmol/L	39.2 ±8.1	37.6±8.5	0.057	28.7±7.0	26.9±7.0	0.025	0.010	0.009
Dimethylglycine, µmol/L	2.0±1.1	2.0±1.2	0.388	1.9±0.7	1.9±0.6	0.995	0.359	0.265
TMAO [3], µmol/L	3.8±1.1	6.1±5.2	0.267	4.4±3.1	5.2±3.3	0.268	0.992	0.729
SAM, nmol/L	101±20	101±20		99±14	98±14		0.937	0.807
SAH, nmol/L	20.0±5.4	20.8±5.4		14.5±2.9	12.3±5.1		0.015	0.002

Data are mean ± SD. N = 10 women and 8 men. [1] P values are according to paired t-test within each sex. [2] Men and women were compared by using ANOVA test. [3] TMAO concentrations were log-transformed before applying t-test. OFTT, oral fat tolerance test; TMAO, trimethylamine N-oxide. .

Supplemental Table 5. Plasma concentrations of choline metabolites, glucose, and lipids at baseline and after applying OGTT according to sex.

	In men			In women				
Plasma biomarkers	Baseline OGTT (fasting)	After OGTT (2 hrs)	*p* [1]	Baseline OGTT (fasting)	After OGTT (2 hrs)	*p* [1]	P [2] between sex (baseline)	*p* [2] between sex (after OGTT)
Free choline, µmol/L	11.1 ±2.4	12.4±2.8	0.011	9.2±2.3	10.0±2.2	0.007	0.111	0.064
Betaine, µmol/L	42.7±7.1	41.7±7.4	0.679	27.2±5.1	27.6±5.9	0.575	<0.001	0.001
Dimethylglycine, µmol/L	2.1±1.3	1.7±1.1	0.027	2.0±0.4	1.7±0.5	0.114	0.798	0.898
TMAO [3], µmol/L	3.8±1.5	3.3±1.2	0.006	4.1±2.6	3.7±2.7	0.113	0.752	0.697
SAM, nmol/L	105±23	101±20	0.236	101±13	92±13	0.101	0.634	0.244
SAH, nmol/L	18.6±6.3	16.9±5.4	0.149	12.9±2.5	12.9±4.7	0.996	0.017	0.112

Data are mean ± SD. N = 10 women and 8 men. [1] P values are according to paired t-test within each sex. [2] Men and women were compared by using ANOVA test. [3] TMAO concentrations were log-transformed before applying t-test. OGTT, oral glucose tolerance test; TMAO, trimethylamine N-oxide.

Supplemental Table 6. Post-hoc calculations of sample size needed to show significant differences in TMAO according to the mean and SD observed in the present study. The power calculations were performed using G*Power 3.1.9.2

	**Pre OFTT**	**Post OFTT**
TMAO, µmol/L	4.1 ± 2.3	5.6 ± 4.1

t tests - Means: Difference between two dependent means (matched pairs)

Input: Tail(s) = Two

Effect size dz = 0.5077237

α err prob = 0.05

Power (1-β err prob) = 0.80

Output: Noncentrality parameter δ = 2.9166506

Critical t = 2.0369333

Df = 32

Total sample size = 33

Actual power = 0.8073483

	**Pre OGTT**	**Post OGTT**
TMAO, µmol/L	4.0 ± 2.1	3.5 ± 2.1

t tests - Means: Difference between two dependent means (matched pairs)

Input: Tail(s) = Two

Effect size dz = 0.8694009

α err prob = 0.05

Power (1-β err prob) = 0.80

Output: Noncentrality parameter δ = 3.1346695

Critical t = 2.1788128

Df = 12

Total sample size = 13

Actual power = 0.8202666

Supplemental Data Files

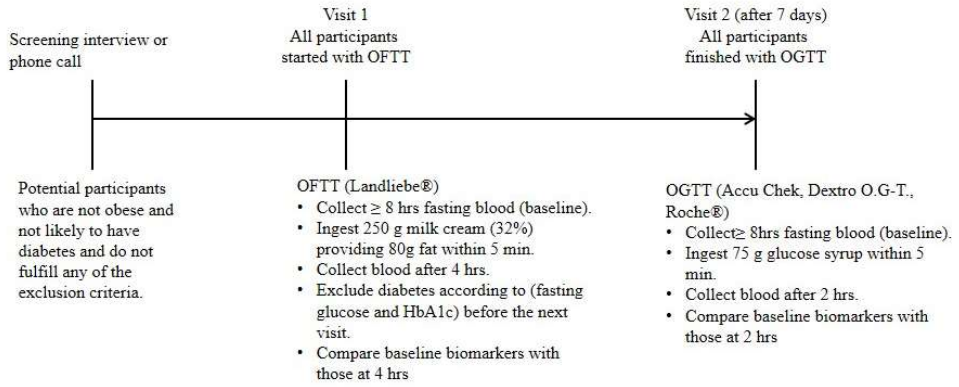

Supplemental Figure 1. Study flow diagram.

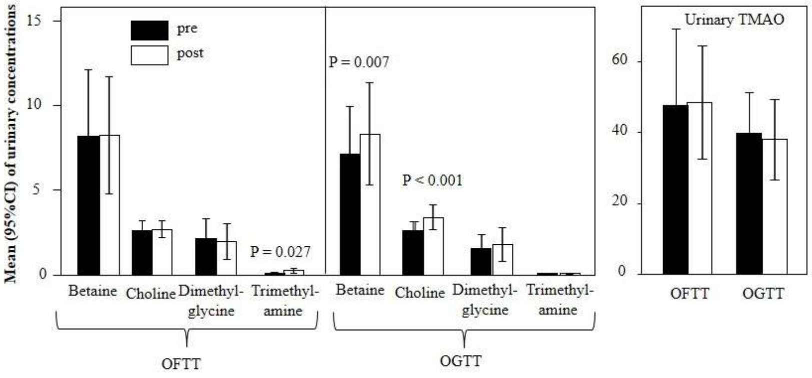

Supplemental Figure 2. Mean and 95%CI of urinary concentrations of the metabolites (all are μmol/mmol creatinine) before and after OFTT and OGTT. Urinary trimethylamine increased from baseline after OFTT, and urinary betaine and choline increased after OGTT compared with fasting concentrations. Urine samples were collected before OFTT and OGTT, 4 hours after OFTT, and 2 hours after OGTT. Concentrations of betaine and choline in urine were higher after OGTT as compared with those after OFTT.

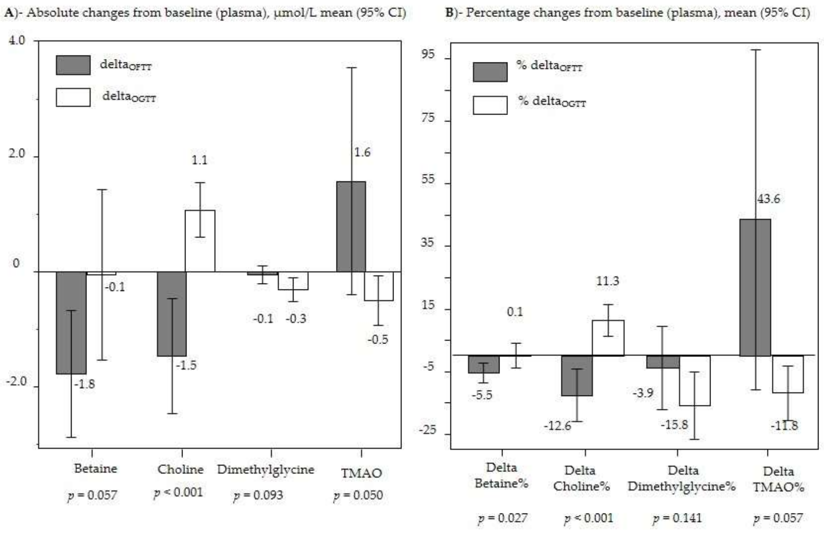

Supplementary Figure 3. A)- DeltaOFTT and deltaOGTT of plasma concentrations of betaine, choline, dimethylglycine, and TMAO. The delta was calculated as (post-test concentrations − pre-test concentrations). B)- the percentage changes of the metabolic markers from baseline. The percentage changes were calculated as: (post-OFTT levels − pre-OFTT levels)*100/pre-OFTT levels or (post-OGTT levels − pre-OGTT levels)*100/pre-OGTT levels. P values are according to paired t-test**.**

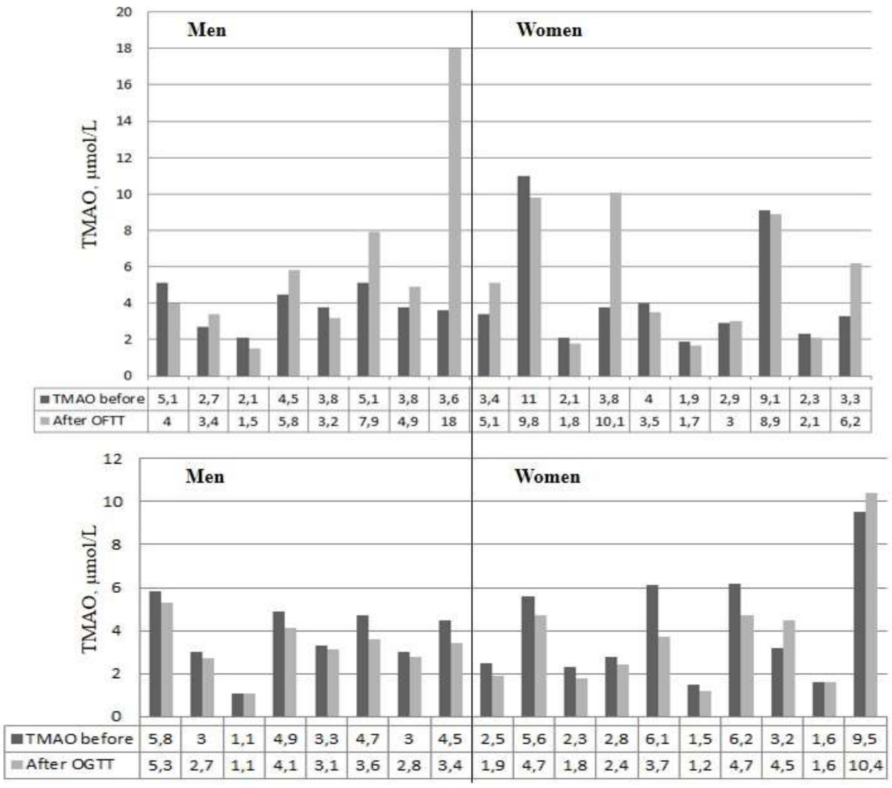

Supplemental Figure 4. Individual TMAO levels (in μmol/L) measured at baseline (before) and after OFTT or OGTT separated according to sex.

Summary post OFTT	Summary post OGTT
↓ Choline	↑ Choline
↓ Betaine	= Betaine
= Dimethylglycine	↓ Dimethylglycine
↑ Betaine/choline	↓ Betaine/choline
↑ TMAO (↑n=9 + ↓n=9)	↓ TMAO (↓n=15 + ↑n=3)
↓ Choline/TMAO	↑ Choline/TMAO
↓ Glucose	= Glucose
= SAM	↓ SAM
↑ Triglycerides	
↑ Urinary trimethylamine	= urinary trimethylamine
= Urinary betaine	↑ Urinary betaine
= Urinary choline	↑ Urinary choline

Supplemental Figure 5. Summary of the significant changes (or tendencies up to ≤ 0.10) in plasma metabolites after applying oral fat tolerance test and oral glucose tolerance test.
